# A Machine Learning Approach for the Identification of a Biomarker of Human Pain using fNIRS

**DOI:** 10.1038/s41598-019-42098-w

**Published:** 2019-04-04

**Authors:** Raul Fernandez Rojas, Xu Huang, Keng-Liang Ou

**Affiliations:** 1 University of Canberra, Human-Centred Research Centre, Canberra, 2617 Australia; 2 Taipei Medical University Hospital, Department of Dentistry, Taipei, 110 Taiwan; 3 Taipei Medical University Shuang Ho Hospital, Department of Dentistry, New Taipei City, 235 Taiwan; 4 Health Sciences University of Hokkaido, School of Dentistry, Hokkaido, 061-0293 Japan; 5 Hasanuddin University, Department of Prosthodontics, Makassar, 90245 Indonesia; 6 Universitas Gadjah Mada, Department of Prosthodontics, Yogyakarta, 55281 Indonesia; 7 Ching Kuo Institute of Management and Health, Department of Oral Hygiene Care, Keelung, 203 Taiwan; 8 3D Global Biotech Inc., New Taipei City, 221 Taiwan; 9 Present Address: School of Engineering and Information Technology, University of New South Wales, Canberra, 2612 Australia

## Abstract

Pain is a highly unpleasant sensory and emotional experience, and no objective diagnosis test exists to assess it. In clinical practice there are two main methods for the estimation of pain, a patient’s self-report and clinical judgement. However, these methods are highly subjective and the need of biomarkers to measure pain is important to improve pain management, reduce risk factors, and contribute to a more objective, valid, and reliable diagnosis. Therefore, in this study we propose the use of functional near-infrared spectroscopy (fNIRS) and machine learning for the identification of a possible biomarker of pain. We collected pain information from 18 volunteers using the thermal test of the quantitative sensory testing (QST) protocol, according to temperature level (cold and hot) and pain intensity (low and high). Feature extraction was completed in three different domains (time, frequency, and wavelet), and a total of 69 features were obtained. Feature selection was carried out according to three criteria, information gain (IG), joint mutual information (JMI), and Chi-squared (*χ*^2^). The significance of each feature ranking was evaluated using three learning models separately, linear discriminant analysis (LDA), the K-nearest neighbour (K-NN) and support vector machines (SVM) using the linear and Gaussian and polynomial kernels. The results showed that the Gaussian SVM presented the highest accuracy (94.17%) using only 25 features to identify the four types of pain in our database. In addition, we propose the use of the top 13 features according to the JMI criteria, which exhibited an accuracy of 89.44%, as promising biomarker of pain. This study contributes to the idea of developing an objective assessment of pain and proposes a potential biomarker of human pain using fNIRS.

## Introduction

Pain itself is a biomarker of many diseases, injuries, or emotional stress and serves as warning mechanism for the brain to act against something wrong in the body^1^. For example, chest pains may be an indicator of a heart disease or headaches may be a sign of stress or fatigue, therefore pain sensation is a warning to avoid potentially dangerous situations. This pain mechanism is a vital function of the human body and is based on the peripheral nervous sytem (PNS), the spinal cord and the brain^2^. Bornhovd *et al*.^3^ described the tasks that the pain processing system serves to prevent potentially life-threatening conditions: collect and analyse nociceptive sensory input, shift the focus of attention towards pain processing, maintain pain-related information in working memory, have prompt communication with the motor system to avoid further damage, and memory-encode the problem to avoid future damage. All of these actions induced by the human pain mechanism have obvious importance for survival.

In clinical practice, there are two main methods for the estimation of pain in patients: self-reports and clinical judgment. Self-reports (numeric and verbal) are the most widely used methods of collecting pain information and regarded as the most accurate^4^. This method relies on a patient’s ability to communicate a self-assessment of pain; visual analogue scales, verbal descriptor scales, numerical rating scales, or the MacGilll pain questionnaire are some examples of subjective metrics of pain. When self-reports are unavailable or unreliable, clinical observations can be used as substitution. However, clinical observations are susceptible to assessment bias^5^; over- or under-estimating the patient’s pain sensation could be fatal for the patient. Therefore, identification of biomarkers to measure human pain is required in clinical practice to improve pain management, reduce risk factors, and contribute to a more objective, valid, and reliable diagnosis.

Some biomarkers have been proposed to measure and identify pain. For instance, salivary cortisol has been used as biomarker for acute pain, however, salivary cortisol variations are not only due to pain-related problems^6^. Cerebrospinal fluid has been reported to identify pain in patients with neuropathic pain and movement disorders^1^. The use of glutamate (an excitatory neurotransmiter) as possible biomarker of fibromyalgia (chronic widespread of muscle pain) showed a correlation between pain and the levels of glutamate in the insula during a functional magnetic resonance imaging (fMRI) study^7^. For gastrointestinal disorders, the use of pharmacological agents such as fentany and octrotide have showed variability in sensory end points, however, there is no clear evidence of an effective biomarker of visceral pain^1^. These examples show that biological variables can be used as potential biomarkers of pain.

Neuroimaging methods are increasingly used to assess pain-related information in the brain and gain further insights into the neural signature of human pain. In these methods, activation of brain areas related to the processing of pain can be identified in response to noxious stimuli^8^. One of these neuroimaging methods is functional near-infrared spectroscopy (fNIRS), which facilitates (in a non-invasive manner) the measurement of brain activity by reading cerebral haemodynamics and oxygenation^9^. This technique has been widely used in diverse clinical and experimental settings, offering advantages over other technologies (fMRI, EEG, PET) such as, better temporal and spatial resolution, less exposure to ionising radiation, safe to use over long periods and repeatedly, less expensive, easy to use, and portable^10^.

In this context, machine learning has been fundamental for the success of neuroimaging techniques in the study of pain^11^. Machine learning is used to better interpret the complexity of pain by revealing patterns in clinical and experimental data, and by obtaining usable information that is essensential to acquiere new knowledge^12^. In classification problems, machine learning makes use of pain-related data to create a mapping of features (also called: variables, predictors, or attributes) and to learn a signature of pain (or class), this new knowledge can be applied on new data to identify or predict the type of pain it belongs to. For instance, Brown *et al*.^13^, in an fMRI study, used the support vector machine (SVM) algorithm to classify painful and non-painful experimental stimuli with 81% accuracy. In an EEG study, Gram *et al*.^14^ predicted the analgesic effect of a drug treatment during rest and pain session using SVM with an accuracy of 72%. In another EEG-based study, Huang *et al*.^15^ used a Naive Bayes classifier to predict low and high pain induced by laser-evoked potentials (LEPs) with an accuracy of 86.3%. The results of these neuroimaging studies using machine learning demonstrates that classification and identification of different types of human pain is plausible.

Many factors influence human pain perception and appropriate measurement techniques must be chosen to capture these influences. In general, finding a good data representation that can simplify the most emblematic patterns in the data is a task-specific problem^16^; in other words, it facilitates the process of getting answers from the data. Therefore, engineering a good set of features that can discriminate the learning problem is a core part of machine learning and a prerequisite for obtaining good performance in any learning task^17^. In order to obtain a good data representation, features should be informative, independent, and simple; they make the later stages in the learning process easy and the learning model more robust to solve the given problem. Bad features, on the other hand, might not produce the same level of success while requiring more complex models, which are generally difficult to understand^18^.

Therefore, to construct adequate features that can extract informative characteristic from noxious stimulation, features should possess the following properties: their spatial, temporal, and spectral characteristics should be representative for a subject or a group of subjects^19^; they should reflect changes during neural activation; they should present a degree of stable correlation with the pain sensation; their value should be similar for stimuli in the same category and different for stimuli in different categories^20^; they should present the lowest possible computational complexity, thus, the feature can be implemented at low cost and in real time. Unfortunately, the literature about optimal features (and its construction) for the classification of different painful stimulus is limited^21^. Therefore, it would be important for the scientific community, to explore different types of features and show their performance for the classification process of different levels of pain.

This paper proposes a biomarker of pain using fNIRS and machine learning. With that in mind, we collected pain information from 18 subjects using the quantitative sensory testing (QST) method for thermal stimulation (heat and cold) and corresponding intensity (low and high). We used this data to extract temporal-, spectral-, and wavelet-based features. We utilised feature selection techniques (filter methods) for the identification of possible biomarkers, and identify the best feature subset (biomarker) by comparing the performance of three well-established classifiers (linear discriminant analysis (LDA), support vector machines (SVM) and K-nearest neighbour (K-NN)) on the fNIRS data. Future research is needed to evaluate our biomarker on independent datasets for pain diagnosis in real-life scenarios.

## Results

### Thermal tests

**Figure 1:**
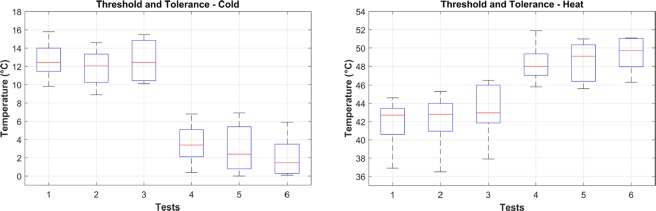
Thermal threshold and tolerance levels perceived by the participants after cold (left panel) and heat (right panel) stimuli. Horizontal red lines are the median values across all participants for each test. Pain threshold (tests 1–3) and pain tolerance (tests 4–6).

Thermal threshold and tolerance of pain perception were obtained following the thermal test in the quantitative sensory testing (QST). By obtaining the QST thermal tests, we aimed to minimize the subjective nature of self-reported pain scores and to apply a set of standard stimuli to all the participants. In this way, the obtained measurements are based on temperature readings to label a measurement as low pain (threshold) or high pain (tolerance) and not on self reports. The measured (averaged) values obtained from each experiment are shown in Fig. 1.

The two plots show the threshold and tolerance temperatures of cold (Fig. 1, left panel) and heat (Fig. 1, right panel) stimuli across all participants. The first three measurements in each plot refer to the pain threshold while the last three measurements refer to the pain tolerance. The median temperature values (horizontal red lines in Fig. 1) in which participants first perceived pain (pain threshold) from cold (12.45 ± 1.97, 12.05 ± 1.93, 12.45 ± 2.22 °*C*) and heat (42.70 ± 2.44, 42.80 ± 2.75, 42.95 ± 2.92 °*C*) clearly presented differences from the highest intensity of pain (pain tolerance) the participants could take from cold (3.40 ± 2.07, 2.40 ± 2.71, 1.45 ± 2.12 °*C*) and heat (48.00 ± 1.92, 49.10 ± 2.11, 49.70 ± 1.82 °*C*). The obtained values were used to identify the classes (and labels) of the database according to corresponding type of pain (heat/cold) and level of pain (low/high).

### Domain-based classification

The classification methods were first applied to the extracted features without performing any feature selection to obtain baseline data. This allows us to obtain reference values from each domain separately and in a combination of all features. The classification was carried out using the top-three nearest neighbours and using the best hyperparameters for both SVMs. Table 1 presents the accuracy of the defined features in each domain. The results in each separated domain showed that the best accuracy was obtained using frequency-based features (84.44%) and the wavelet-based features (84.72%), using the Gaussian SVM in both cases; while for the features in time domain, the best accuracy (73.98%) was achieved by the nearest neighbour classifier. Using a combination of all the domain representations (69 features) produced the highest accuracy (88.41%) among all representations using the Gaussian SVM. These results showed that most of the features are not linearly separable and using a Gaussian kernel improves the accuracy. This is also demonstrated in the results of the linear classifiers (LDA and Linear SVM) with the worst accuracy in the time and frequency domain.

**Table 1:** Accuracy of the seven classifiers using features only from each domain separately.

Classifiers	Domain Representations			
	Time (9)	Frequency (23)	Wavelet (37)	All (69)
LDA	40.40	65.15	64.64	81.33
1-NN	**73.98**	81.06	76.5	86.38
3-NN	66.91	74.74	71.71	84.59
5-NN	62.87	70.7	64.89	80.55
Linear SVM	41.11	65.55	69.72	81.88
Gaussian SVM	71.38	**84.44**	**84.72**	**88.41**
Polynomial SVM	68.05	80.55	77.50	70.27

Numbers in parenthesis represent the number of features from each domain used in the classification process. The results are presented in percentages.

### Classification of ranked features

**Figure 2:**
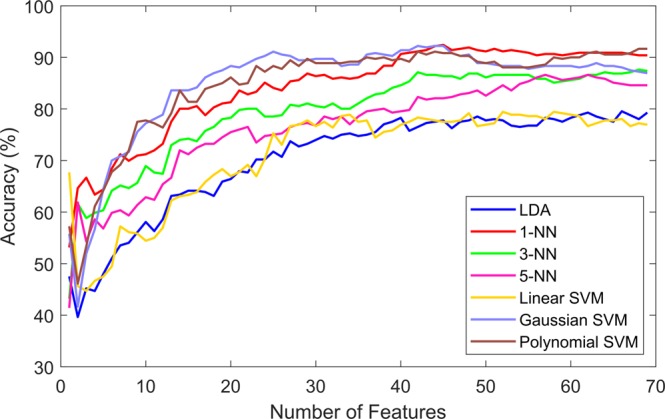
Classification results by seven different learning models using the ranked features according to the information gain (IG) criterion.

**Figure 3:**
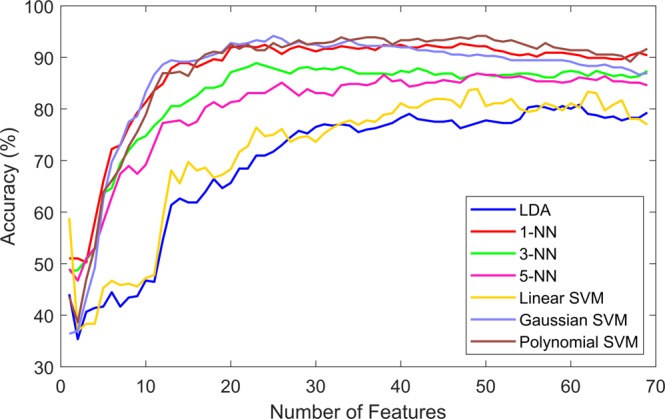
Classification results by seven different learning models using the ranked features according to the joint mutual information (JMI) criterion.

**Figure 4:**
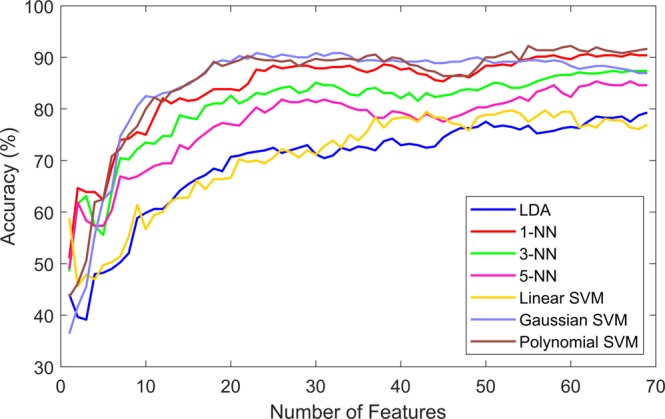
Classification results by seven different learning models using the ranked features according to the Chi-squared (Chi-2) criterion.

After applying the feature selection methods, the features were ranked according to each particular criteria. The expectation was that the classification accuracy would improve if the less relevant and more redundant features were not included in the classification process. The three ranking criteria were evaluated, the evaluation process was done by including one feature at a time in the classification process and observing the accuracy for each particular subset. Figures 2–4 show the classification accuracy for each subset. For the information gain (IG) heuristic, the best result was achieved by the top 45 ranked features with an accuracy of 92.42% using the 1-NN classifier. The best results for the joint mutual information (JMI) ranking criterion was 94.17% with the Gaussian and Polynomial SVM using the top 25 features and top 49 features, respectively. The chi-squared (*χ*^2^) method showed the lowest accuracy out of the three heuristics, the top 56 ranked features presented 92.22% accuracy using the Polynomial SVM. Table 2 presents the highest accuracy for each classier using the three ranking criteria, in parenthesis the number of features used to achieve that particular performance is shown. These results showed that 1-NN, the Gaussian SVM, and Polynomial SVM produced the highest accuracy.

**Table 2:** Accuracy of the classifiers using the ranked features. Only the results with the highest accuracy are presented.

Classifiers	Accuracy (Number of features)		
	IG	JMI	Chi-2
LDA	79.54 (66)	80.80 (61)	79.29 (69)
1-NN	**92.42** (45)	92.67 (47)	90.65 (62)
3-NN	87.62 (68)	88.88 (23)	87.37 (63)
5-NN	86.61 (57)	86.86 (49)	85.35 (63)
Linear SVM	79.44 (52)	83.88 (49)	79.72 (53)
Gaussian SVM	92.22 (42)	**94.17** (25)	90.83 (23)
Polynomial SVM	91.66 (68)	**94.17** (49)	**92.22** (55)

The number of features used to achieve the highest accuracy is presented in parenthesis. The accuracy is displayed in percentages (%). For example, IG using LDA produces an accuracy of 79.54% using 66 features.

Some of the aims of using feature selection are: decreasing the computational time, reducing complexity, and improving accuracy of the learning models. In order to accomplish these goals, it is necessary to obtain the highest possible accuracy while using the lowest number of features. Results presented in Table 2 show that although some classifiers (e.g Polynomial SVM and 1-NN) produced high accuracy, these needed a large number of features. For instance, the 1-NN classifier with the IG ranking used 45 features to produce an accuracy of 92.42%. Another example is the polynomial SVM with the Chi-squared ranking using 55 features to obtain an accuracy of 92.22%. In these particular examples, these classifiers do not meet the requirements for the purpose of feature selection. Therefore, finding the classifiers that produce high accuracy with the minimum number of features is desirable.

From Table 2 a classifier that produced high accuracy with substantially less features than the rest is the Gaussian SVM. A clear example is the comparison between the performance of the Gaussian and polynomial SVM classifiers using the JMI ranking, both resulted in 94.17% accuracy; however, the Gaussian SVM needed only 25 features compared to the 49 features used by polynomial SVM. Another example is the accuracy presented by the Gaussian SVM (90.83%) and the Polynomial SVM (92.22%) with the ranking produced by the Chi-squared method, using 23 and 53 features, respectively; again the Gaussian SVM uses a lesser number of features while producing nearly the same accuracy. In these cases, it is more efficient to use the Gaussian SVM because it will be faster and less complex than the Polynomial SVM and the 1-NN while using only half of the number of features.

### Best feature subset

The Gaussian SVM classifier showed a sound performance with all the feature selection methods and using less features than other classifiers. In addition, the Gaussian SVM exhibited the highest accuracy (94.17%) in this analysis with the joint mutual information (JMI) technique. The results presented in Fig. 3 also show that the Gaussian SVM exhibits a stable response with different numbers of features. Most importantly from Fig. 3, using the Gaussian kernel with only the top 13 features produces an accuracy of 89.44%, which is higher than the highest initial accuracy (88.41%) produced with the 69 features in Table 1. These top 13 features are summarized in Table 3. The construction of these features represent a multidimensional approach to potentially find the best attributes from each domain to characterize human pain. Therefore, we propose these 13 features as potential biomarker of human pain because they produce good classification accuracy, represent a small feature space, and the features are mostly generated in the expected activation bands (VLFO and LFO).

**Table 3:** Top 13 features ranked by the joint mutual information (JMI) method, producing an accuracy of 89.44% using the Gaussian kernel SVM.

Ranking	Name	Description	Domain,	Band	Frequency
1	timepeak	Time to highest peak	Time	—	—
2	F5	Fourier coefficient	Frequency	VLFO	0.055 *Hz*
3	W5	Wavelet coefficient	Wavelet	LFO	0.113 *Hz*
4	W29	Wavelet coefficient	Wavelet	VLFO	0.0214 *Hz*
5	varvl	Variance of Fourier coefficients	Frequency	VLFO	0.01–0.08 *Hz*
6	vwvl	Variance of wavelet coefficients	Wavelet	VLFO	0.01–0.08 *Hz*
7	mean	Time mean	Time	—	—
8	W11	Wavelet coefficient	Wavelet	VLFO	0.0746 *Hz*
9	F11	Fourier coefficient	Frequency	LFO	0.122 *Hz*
10	vwl	Variance of wavelet coefficients	Wavelet	LFO	0.08–0.15 *Hz*
11	W25	Wavelet coefficient	Wavelet	VLFO	0.0283 *Hz*
12	F7	Fourier coefficient	Frequency	VLFO	0.077 *Hz*
13	W21	Wavelet coefficient	Wavelet	VLFO	0.0373 *Hz*

## Discussion

In this study we presented a potential biomarker of human pain based on measurements using functional near-infrared spectroscopy (fNIRS). The pain recognition task showed that the Gaussian kernel support vector machine exhibited the best accuracy (94.17%) with only 25 features among all the classifiers, which represented a significant improvement from the initial (88.41%) classification results using a combination of all 69 features. In addition, this study proposes the use of the top 13 features (presented in Table 3) generated by the joint mutual information (JMI) method as possible biomaker of human pain using fNIRS.

Although there are studies of pain detection using neuroimaging methods, these studies focused on two conditions only^13,14,22,23^. These conditions are based on pain or no-pain (a binary classification) using a single type of stimulation (e.g., cold, heat, or electrical). These studies did not propose models that can differentiate multiple signatures of pain. This is particularly a problem since in the human body, pain can have different origins (e.g., peripheral, visceral, emotional, phantom pain), different intensities and durations. Each type of pain is detected and carried to the central nervous system by different sensory receptors^21^. Therefore, proposing machine learning models, which are able to differentiate multiple signatures of pain and at different intensities, would be more valuables for realistic scenarios. This is critical for patients unable to speak (e.g., in coma or with advanced dementia) and when the source of pain is not evident.

**Figure 5:**
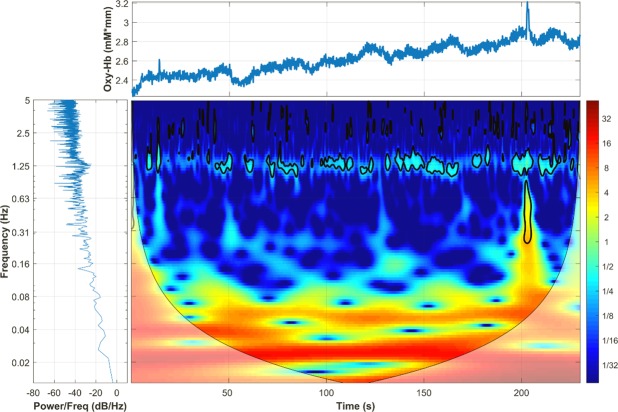
Time-frequency analysis (bottom-right panel) of a raw HbO signal using the wavelet transform. Heartbeat signal can be seen in the frequency of ~1.25 *Hz*, it is exhibited as a large peak in the frequency spectrum (bottom-left panel) and affects the data during the whole experiment as observed in the wavelet domain. The effect of a moving artefact is also observed after the last stimulus (after time 200 sec) in the temporal graph (top panel), which affects several frequency bands (only observed in the wavelet domain).

Most of the features in the proposed biomarker are from the wavelet domain. It is well know, that a major disadvantage of frequency analysis, in particular the Fourier transform, is that the temporal information is lost in the frequency domain. Similarly, a disadvantage of time-domain analysis is the impossibility to access frequency components in the fNIRS signals. Wavelet analysis remedies these two drawbacks by producing a time-frequency representation of the original fNIRS signals^19^; this can be seen in Fig. 5, where two domain-specific phenomena can be clearly visualised. For instance, in the time domain (top panel) it is possible to observe a large motion artefact after the 200 *sec* mark, an event that cannot be easily identified (at least without additional analysis) in the frequency domain. Similarly, a strong physiological signal such as the subject’s heartbeat can be clearly seen in the frequency domain (bottom-left panel) as a large peak in the frequency of ~1.25 *Hz*, while in the time domain it is not easily observed. However, in the wavelet domain these two events can be identified simultaneously. First, the motion artefact is easily detected in the same time period (after 200 *sec*) and also it is possible to see the frequencies (~0.20–1.25 *Hz*) affected by this type of noise. Second, the heartbeat can be clearly detected in the same frequency as observed in the Fourier analysis and it is also clear that this physiological signal affects the HbO signal during the whole experiment; therefore, this frequency could be filtered out. Therefore, a clear advantage of wavelet analysis is the ability to obtain information from time and frequency simultaneously.

In the classification task, three classifiers showed better results than the rest, these were: the 1-NN, Gaussian SVM, and Polynomial SVM. However, the Gaussian SVM exhibited the highest accuracy with lowest number of features. One of the assumptions to use a Gaussian kernel SVM is that the data is not linearly separable, and therefore, the use of a kernel to map the data to a higher-dimensional feature space where the data can be separable is recommended^24^. This can be also observed in the performance of the linear classifiers (LDA and SVM), which had the lowest accuracy (refer to Table 2). These results suggest that most of the features do not follow a normal distribution making the data separation more difficult with observations that are not linearly separable, and thus, the use of a method such as Gaussian SVM will produce a better perfomance for such type of data. However, this is not always the case, for example, in cases with larger numbers of features (~thousands) a linear SVM will suffice, and mapping the data to a higher dimension space does not improve the performance^25^; in such cases, a complex model such as a Gaussian SVM may lead to overfitting and this model is also more expensive to train due to the number of hyperparameters to tune. Based on this observations, the use of an assemble classifier to learn both normal and non-normal features might produce better results.

The identified features represent the best subset for the thermal tests defined for this particular study. It would be ideal to explore the effectiveness of the proposed biomarker with other types of experimental noxious stimuli (e.g. mechanical, electrical, or laser-evoked) and in real scenarios. In particular, to study features that can identify the frequency bands where different stimuli can be better isolated for its recognition and analysis. For example, an electrical stimulus might cause a particular reaction in a given frequency band, and a mechanical stimulation (e.g, pin prick) might produce a different response in another frequency band. By examining different pain conditions, new features might be identified for specific frequency bands. It might be possible to assign a specific frequency band to a group of conditions with a similar frequency response. In a study using fNIRS by Lee *et al*.^26^, the activation patterns for pain and itchy stimulation were similar but with distinct delayed responses; this might suggest different frequency response for these two stimuli. Therefore, it would be valuable to identify the spectrum response of different noxious stimuli and define specific features in their corresponding frequency bands that can be used to identified and isolate different pain conditions.

A limitation in this study is the lack of control for any skin blood flow contributions and intracerebral haemodynamics to the fNIRS signals. Recent studies have highlighted the issue that fNIRS signals encompass not only haemodynamic fluctuations due to neurovascular coupling but also due to skin blood flow and task-related systemic activity of cortex^27,28^. Extracerebral haemodynamics originate in shallow tissue (e.g., scalp), the area in which NIR light travels before reaching the cerebral cortex, and thus, contributions to the observed fNIRS signals are expected. Similarly, intracerebral haemodynamics are caused by systemic physiological interference that might resemble true task-related cortical activities, these physiological variables are mainly associated with cardiac pulsations, respiration, arterial blood pressure, and activity in the autonomic nervous system^27^. In our case to avoid these two potential confounders (extra- and intracerebral): first, the observed activation was measured in areas known to be involved in pain processing, which suggests that the fNIRS signals reflect mainly localized cortical vascular dynamics; second, PCA was used to identify those principal components that account for most of the variance while in resting state, and then reduce these PCs (potentially both, extra- and intracerebral confounders) in the stimulus data^28^; and third, this analysis was mainly focus on frequency bands that are only related to the expected period of response and therefore avoiding those bands related to heartbeat (~1 *Hz*) and respiration (~0.3 *Hz*). However, we acknowledge that the followed de-noising procedure might not be enough to reduce such sources of haemodynamic interference, and other techniques such as using short-separation channels to reveal scalp blood flow, or employing additional instruments to measure physiological signals would be desirable to regress out these undesired signals.

fNIRS has demonstrated to be a method that has potential for the assessment of pain. fNIRS is a technique capable of identifying cortical heamodynamic changes in response to chemical, temperature, and pressure noxious stimuli^9,21^. In addition, this research demonstrates that the use of fNIRS in combination with machine learning techniques is a powerful tool for the assessment of pain in experimental settings. fNIRS possesses advantages over PET or fMRI for use in more realistic clinical settings, e.g., it is less expensive and of small size. Certainly, the findings provided in this study advance knowledge in pain assessment using fNIRS as the method of diagnosis and represent a step closer to developing a physiologically-based diagnosis of human pain that would benefit not only vulnerable populations who cannot self-report pain, but also the whole population.

This paper presents the following novelties with respect to the current literature: (1) It presents a classification model able to discriminate between multiple pain signatures and at different intensities. This is a substantial innovation in the field since other studies have only focused on pain and no-pain models. (2) It presents a study of different feature extraction techniques in different domains (time, frequency, and wavelet). (3) It also identifies a subset of 13 features with an accuracy of ~90% as potential biomarker of pain using fNIRS data. To the best of our knowledge, there is no other paper in the current literature that studies, compares, and proposes a biomarker of pain using fNIRS. (4) Most importantly, this paper presents a framework to quantify multiple types of pain using fNIRS. We describe our procedure from data taken, pre-processing, feature extraction, feature selection, classification, and biomarker presentation. This is particularly important since this study aims to show evidence of reproducible research, and the presented framework can be easily generalized to larger studies from varied fNIRS recording protocols.

Future work should include more advanced methods for feature selection, classification, and feature extraction. Thus, an optimal approach to the assessment of human pain can be determined. In addition, the proposed biomarker should be evaluated on new datasets before it is used in clinical applications, and collecting more data from different populations (e.g., age, ethnicity) should also be considered to achieve a more robust learning model. Despite the limitations, this study serves as a baseline for future research in biomarkers identification for pain assessment using imaging methods. Finally, this study contributes to the idea of developing an objective assessment of pain that would benefit patients unable to communicate (non-verbal) pain information (e.g., elderly with advance dementia, patients with intellectual disabilities, or pre-verbal infants). This study suggests a potential biomarker of pain using fNIRS and machine learning.

## Methods

### Participants

Eighteen right-handed volunteers (three females) were considered in the study, mean age ± standard deviation (31.9 ± 5.5). All participants were right-handed to avoid any variation in functional response due to lateralisation of brain function. No participants reported a prior history of neurological or psychiatric disorder, a current unstable medical condition, or under medication at the time of testing. For study participation, written informed consent was obtained from all participants prior to initiation of the experiments. Throughout the experiments, the participants were instructed to keep their eyes closed^29^. Procedures and methods for this study followed the guidelines accepted by the Declaration of Helsinki. This research study was approved by full-board review process of the Taipei Medical University’s Joint Institutional Review Board under Contract Number 201307010.

### Pre-processing

Raw fNIRS data is generally contaminated by different sources of noise and pre-processing is required. Electrical noise in fNIRS data and cardiac pulsation (~1 *Hz*) was canceled by a finite impulse forth-order low-pass filter with a cut-off frequency of 0.16 *Hz*; this cut-off frequency helpped maintain the very-low frequency oscillations (VLFO)(0.01–0.08 *Hz*) and low frequency oscilation (VLO)(0.08–0.15 *Hz*) bands^30^. Then, motion artifacts were removed following a wavelet de-noising procedure^31^. In order to reduce extracerebral haemodynamics (scalp) and systemic variables^27^ (e.g. blood pressure, autonomic nervous system acitivty) existent in our data, we used principal component analysis (PCA) to identify and delete those components representing spurious signals^28^.

### Feature extraction

A feature can be defined as a representation of data, which can be binary, categorical or continuous^16^. Features can be defined in many ways and, in general, features are a simplification of the most representative pattern in the raw data. Feature extraction (or feature engineering) is the process of identifying the pertinent signal characteristics (attributes) from extraneous content and representing them in a compact and/or meaninful form, amenable to interpretation by a human or computer^19^. Feature extraction occurs after signals have been acquired and cleaned from noise, and it gathers the attributes that are used to solve the learning problem.

The number of studies in classification of experimental pain is small and direct comparisons with these studies is limited^21^. In addition, these studies used a small feature space to characterize the sample data and build a binary prediction (pain or no pain) with different success. Therefore, in this research, other similar applications in BCI and biomedical time-series analysis are used to construct a better representation from the fNIRS data. Most features in neural applications use spatial, temporal, and spectral analysis of brain signals of multiple channels and in different time points^19^. This set of simultaneously computed features is described as feature vector.

In this particular study, an important characteristic of the stimulation experiment is the stimulation intensity and the stimulus periodicity. Thus, the expectation is that features that could reflect these types of characteristics may produce better results. Identifying these attributes from the fNIRS data would be desirable to make the classification task simple and approaching high classification accuracy^32^. Each extracted feature was computed for each of the 24 channels, and with each channel containing its own vector. The idea behind this methodology is obtaining a multidimensional analysis which broadens the scope of pain to achieve the best representation of human pain. The feature extraction techniques are organized in three groups: time domain, frequency domain, and wavelet domain.

#### Time domain

The haemodynamic response after each stimulus produces a positive response of HbO concentration. This response is referred to as the activation curve which generally is initiated by a small dip, followed by an increase of HbO to reach its maximum (peak) response, and then return to baseline. This response can be used to characterise different haemodynamic response (i.e., cold, heat). The extracted features in time domain (a total of 9 features) from the HbO activation curve are: mean, variance, skewness, kurtosis, peak amplitude, slope, area under the curve (AUC), time to peak, and root mean squared (RMS).

#### Frequency domain

Fourier analysis provided a mapping of the HbO signal to the frequency domain. This projection exposes the spectral content of the signal in terms of the sum of its projections onto a set of sine or cosine functions. We used the power spectrum density of the HbO signal to divide the original signal into frequency bands. In specific, we were interested in two bands: very-low frequency oscillations (VLFO, 0.01–0.08 *Hz*) and low frequency oscillations (LFO, 0.08–0.15 *Hz*)^30^; these bands correspond to the period of the stimulation task and the main energy distribution is expected in these frequencies. The extracted features (a total of 23 features) in the frequency domain are: Fourier coefficients from the VLFO (8 features) and LFO (7 features) bands, energy spectrum’s mean in each band (2 features), the variance of the energy spectrum in each band (2 features), maximum energy value in each band (2 features), and the frequency of the maximum energy value in each band (2 features).

#### Wavelet domain

The wavelet representation was done by the continuous wavelet transform (CWT). Wavelet analysis provides a detailed description of the power spectrum of the signal in terms of both time and frequency domain^8^. The levels of decomposition are selected according to the VLFO and LFO bands. The features extracted (a total of 37 features) in the wavelet domain are: wavelet coefficients from the VLFO (21 features) and LFO (9) bands, mean of the absolute values of the coefficients in each band (2 features), the variance of the wavelet coefficients in each band (2 features), the wavelet power spectrum from each frequency band (2 features), and the absolute mean ratio between the mean values of the VLFO and LFO band (1 feature). A summary of all defined features is presented in Table 4.

**Table 4:** Summary of defined features (69) from each HbO signal in time, frequency and time-frequency (wavelet) domains.

Number of Features	Name	Symbol	Definition	Domain
1	Mean	*μ*_*t*_	${\mu }_{t}=\frac{1}{N}{\sum }_{n=1}^{N}\,HbO[n]$	Time
1	Variance	*Var*_*t*_	$Va{r}_{t}=\frac{1}{N-1}{\sum }_{n=1}^{N}\,|HbO[n]-{\mu }_{t}{|}^{2}$	Time
1	Skewness	*Sk*_*t*_	$S{k}_{t}=\frac{1}{N-1}{\sum }_{n=1}^{N}\,{(\frac{HbO[n]-{\mu }_{t}}{\sigma })}^{3}$	Time
1	Kurtosis	*Kur*_*t*_	$Ku{r}_{t}=\frac{1}{N-1}{\sum }_{n=1}^{N}\,{(\frac{HbO[n]-{\mu }_{t}}{\sigma })}^{4}$	Time
1	Peak	*Max*_*t*_	Maximum value of HbO	Time
1	Slope	*Kur*_*t*_	*y* = *β*_1_*x*	Time
1	Area Under Curve	*AUC*_*t*_	${\int }_{a}^{b}\,f(HbO)dx$	Time
1	RMS	*RMS*_*t*_	$RM{S}_{t}=\sqrt{\frac{{\sum }_{i=1}^{N}\,Hb{O}^{2}}{N}}$	Time
1	Time to Highest Peak	*timepeak*	Time to maximum value of HbO	Time
15*	Fourier Coefficients	*F*[*k*]	$F[k]={\sum }_{n=1}^{N}\,HbO[n]{W}_{N}^{(n-\mathrm{1)(}k-\mathrm{1)}}$	Frequency
2*	Mean Frequency	*μ*_*f*_	${\mu }_{f}=\frac{1}{N}{\sum }_{n=1}^{N}\,F[n]$	Frequency
2*	Variance of Fourier Coefficients	*Var*_*f*_	$Va{r}_{f}=\frac{1}{N-1}{\sum }_{n=1}^{N}\,|F[n]-{\mu }_{f}{|}^{2}$	Frequency
2*	Maximum Energy	*Max*_*f*_	Coefficient with the highest value	Frequency
2*	Maximum Frequency	*fmax*_*f*_	Frequency of maximum energy	Frequency
30*	Wavelet Coefficients	*W*_*w*_	$W(S,\tau )=\frac{1}{\sqrt{S}}\int \,HbO(t){\psi }^{\ast }(\frac{t-\tau }{S})$	Wavelet
2*	Mean Wavelet	*μ*_*w*_	${\mu }_{w}=\frac{1}{N}{\sum }_{n=1}^{N}\,W[n]$	Wavelet
2*	Variance of Wavelet Coefficients	*Var*_*w*_	$Va{r}_{w}=\frac{1}{N-1}{\sum }_{n=1}^{N}\,|W[n]-{\mu }_{w}{|}^{2}$	Wavelet
2*	Power Spectrum	*WPS*_*w*_	$WP{S}_{w}={\sum }_{k=0}^{{2}^{j}-1}\,{C}_{j,k}^{2}$	Wavelet
1*	Absolute Mean Ratio	*AMR*_*w*_	*AMR*_*w*_ = |*μ*_*w*_*VLFO* − *μ*_*w*_*LFO*|	Wavelet

Features with (*) means that features are obtained from both, the low frequency oscillation (LFO) and the very-low frequency oscillations (VLFO) bands.

A global representation of the different domains used to generate each feature is presented in Fig. 5. This image combines the tree domains, the raw HbO signal in the time domain is presented in the top panel, the power spectrum describing the distribution of power into frequency components composing the HbO signal is presented in the bottom left panel, and in the bottom right panel the HbO signal is exhibited in a time-frequency representation, time in the *x* axis, frequency in the *y* axis.

### Feature selection

Feature selection can be defined as a process to select a subset of features from the original set of features. Thus, the feature space can be optimally reduced according to a certain evaluation criteria^33^. Common objective criteria are: prediction accuracy, data size, and minimal use of input features to reduce associated costs^34^. The main goal of feature selection is to increase the classification performance by building a more compact feature subset, this is achieved by finding and eliminating those redundant or irrelevant features that have little influence to accurately describe the data.

The feature selection process was used to obtain the most relevant features according to three criteria. These criteria are: chi-squared statistics (*χ*^2^), information gain (IG), and joint mutual information (JMI). The reasoning behind using only ranking methods in contrast to other methods (e.g. wrappers) is that ranking methods evaluate the features independently of any classification model, are computationally simple and fast, and are computed only once^16^; these attributes are desired for clinical applications. In general, using feature selection will make the computational model less complex by using the most relevant features and potentially discharging irrelevant features; it will also enable a faster training process of the machine learning algorithm, thus it reduces the computational cost.

### Classification

The classification stage aimed to compare the effectiveness of the three ranking criteria in improving the accuracy of our classification models. The classification problem was to predict the class label (e.g., 1 = Low-Heat, or 4 = High -Heat) of unknown data points into one of the four categories. For that purpose, the data was randomly split on the subject level in training (13 subjects) and validation (5 subjects). One subset was used to train the classifier and the remaining subset was used to validate the performance of the classifier. The training process was carried out using leave-one-out cross validation (LOOCV) on the subject level. Data from one subject was held as the testing set and the remaining data (12 subjects) was used for training; this process was repeated 12 times, testing on a different subject in each iteration. The trained models were then validated using the remaining five subjects and the evaluations of predictive performance (classification accuracy) are then averaged across the five subjects and reported as the final scores. Classification accuracy was calculated by determining the number of correctly predicted labels divided by the total number of testing samples from each subject (i.e., correct/total).

Three well-established algorithms were compared, the linear discriminant algorithm (LDA), the k-nearest neighbour (K-NN), and support vector machines (SVM) for multi-class classification (one-vs-one voting). The LDA classifier is ideal for real-time applications due to its low computational cost and good results; this method was used as ground truth for the classification task. The K-NN model was parametrised using Euclidean distance as metric of similarity and the best K - neighbours were obtained by searching K from 1 to 20; the top five nearest neighbours were K = 1,3,5,7,9. Similarly, for the SVM models a grid search of parameter C (at 0.1, 1, 10, and 100) for both kernels and Gamma (at 0.001, 0.01, 0.1, and 1) for the Gaussian kernel was completed. Best obtained results, for both kernels parameter C = 1 and parameter Gamma = 0.01 for the Gaussian kernel.

### Experimental paradigm

Pain perceptions were investigated using the quantitative sensory testing (QST) protocol^35^. We defined pain threshold (low pain) as the lowest stimulus intensity at which stimulation becomes painful, and pain tolerance (high pain) as the highest intensity of pain at which stimulus becomes unbearable. The participants were exposed to gradually increasing or decreasing temperatures with a sensory analyzer (Pathway CHEPS, Medoc Ltd., Israel), which delivers heat and cold to the skin with a thermode; the thermode has a contact area of 9.0 *cm*^2^ and a baseline temperature of 32 °*C*. Pain measurements were obtained on the back of the left hand, the participants pressed a button when they experienced pain (threshold test) and highest intensity of pain (tolerance test). The temperature of the thermode, just as it became painful or unbearable was recorded as the thermal pain threshold or thermal pain tolerance, respectively. Research has showed no significant difference in QST between the right and left sides of the body^35^.

**Figure 6:**
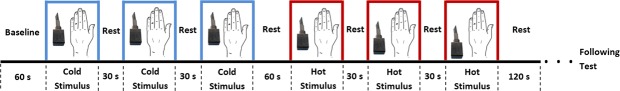
Stimulation paradigm. In this example, pain threshold test was first measured followed by pain tolerance test. In each test, cold and hot stimulus were applied on the back of the hand of each subject. Each stimulus was applied in a random order.

The stimulation protocol consisted of two tests: the thermal pain threshold (low pain) and the thermal pain tolerance (high pain), with a 2-minute rest between each test. Baseline data were measured at rest during the first 60 seconds of the experiments, after that, the stimulation was randomly applied between threshold and tolerance tests. Figure 6 presents an example of the stimulation paradigm. In this example, three consecutive measurements of cold and heat pain thresholds are obtained; 60-second rest between cold and heat detections was applied and a 30-second rest between stimuli was applied. Based on these measurements the fNIRS data were organized into four categories for classification: 1. Low-Cold (low pain), 2. Low-Heat (low pain), 3. High-Cold (high pain), and 4. High-Heat (high pain). These categories (1–4) were used to label the database and used as classes for the classification task.

### fNIRS recording

**Figure 7:**
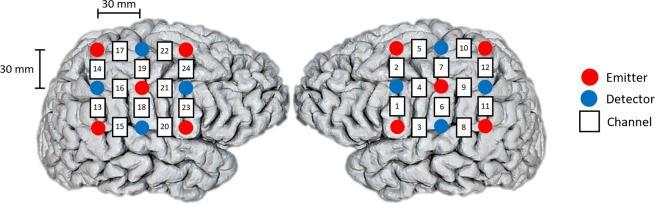
Channel location and configuration. Channel probes were located around the C3 and C4 areas. Source-detector distance was 3 cm.

Brain haemodynamics were acquired using a Hitachi ETG-4000 (Hitachi Medical Corporation, Japan) optical topography system. This system uses near-infrared (NIR) light to investigate cerebral hemodynamics. Two wavelengths of NIR light are used, oxy-hemoglobin (HbO) at 695 *nm* and deoxy-hemoglobin (HbR) at 830 *nm*. The equipment uses a 24-channel cap, configured in 12 channels per hemisphere. According to the EEG 10–20 system, the measuring probes were on the C3 and C4 positions^29^. The head probe has a predefined source-detector separation of 3 cm. Figure 7 presents the probe configuration, channels 1 to 12 sampled the right hemisphere, while channesl 13 to 24 sampled the left hemisphere. In this study, we used only the HbO signals because they exhibit a better signal-to-noise ratio than HbR signals^36^. The sampling frequency used in the experiments was was 10 Hz.
